# Association between High Lipid Burden of Target Lesion and Slow TIMI Flow in Coronary Interventions

**DOI:** 10.3390/jcm11185401

**Published:** 2022-09-14

**Authors:** Subin Lim, Jung-Joon Cha, Soon Jun Hong, Ju Hyeon Kim, Hyung Joon Joo, Jae Hyoung Park, Cheol Woong Yu, Tae Hoon Ahn, Do-Sun Lim

**Affiliations:** 1 Department of Cardiology, Cardiovascular Center, Korea University Anam Hospital, Korea University College of Medicine, Seoul 02841, Korea; 2 Department of Cardiology, Heart and Brain Institute, Chung-Ang University Gwang-Myeong Hospital, Chung-Ang University College of Medicine, Gwangmyeong-si 14353, Korea

**Keywords:** lipid core burden index, near-infrared spectroscopy, thrombolysis in myocardial infarction, percutaneous coronary intervention, intravascular ultrasound

## Abstract

Decreased thrombolysis in myocardial infarction (TIMI) flow is associated with poor clinical outcomes. However, its predictors are not fully known. A combination of near-infrared spectroscopy (NIRS) and intravascular ultrasound (IVUS) could be used to detect lesions at high risk of slow TIMI flow. This study evaluated 636 consecutive patients undergoing target-lesion NIRS-IVUS imaging prior to percutaneous coronary intervention (PCI). The maximal lipid core burden index over 4-mm segments (maxLCBI_4__mm_) per target vessel was calculated. The primary endpoint was the association between maxLCBI_4__mm_ and post-interventional TIMI flow. A high lipid core burden index (LCBI) cut-off point was determined using receiver-operating characteristic analysis. Decreased TIMI flow (TIMI less than 3) occurred in 90 patients and normal TIMI flow in 546 patients. The decreased TIMI flow group showed significantly higher incidence of cardiovascular events (5.6% vs. 1.5%, log-rank *p =* 0.010) in three months of composite events including cardiac death, myocardial infarction, stent thrombosis, and target lesion revascularization. In multivariable analysis, a high LCBI (≥354) was independently associated with slow TIMI flow (OR, 2.59 (95% CI, 1.33–5.04), *p =* 0.005). High LCBI measured using NIRS-IVUS imaging was an independent predictor of decreased post-PCI TIMI flow. Performing PCI for high-LCBI lesions may necessitate adjunctive measures to prevent suboptimal post-PCI reperfusion.

## 1. Introduction

Incomplete restoration of distal blood flow after percutaneous coronary intervention (PCI) is a major cause of adverse clinical outcomes [1,2]. The thrombolysis in myocardial infarction (TIMI) scale is widely used to assess the degree of coronary artery blood flow based on invasive coronary angiographic imaging. Decreased TIMI flow translates to microvascular obstruction of the coronary artery for various reasons, including plaque embolization. The no-reflow phenomenon, defined as a reduction in epicardial flow, may occur in abrupt situations during coronary interventions. Previous studies have reported the incidence of the no-reflow phenomenon to be 2.3–25%, depending on the specific definition [2,3,4]. However, although a decreased TIMI flow is associated with increased cardiovascular events [1,2], independent factors that predict changes in TIMI flow before intervention are yet to be elucidated.

A lipid-rich core plaque is a high-risk feature of vulnerable plaques, which leads to an increased risk of unfavorable outcomes [5,6,7]. Cases of the no-reflow phenomenon after PCI or pre-ballooning on highly lipidic plaques have been reported, some leading to cardiopulmonary resuscitation and mortality [8]. Although intravascular ultrasound (IVUS) and optical coherence tomography (OCT) have evolved to detect high-risk plaques, these modalities are not sufficient to discern the amount of lipid core burden. However, near-infrared spectroscopy (NIRS) is specialized in lipid detection and has the potential to provide quantitative information on plaque vulnerability by assessing lipid burden in terms of the lipid core burden index (LCBI) [9,10].

In this context, this study aimed to identify the predictors of decreased TIMI flow in patients undergoing coronary intervention based on the association between the lipid core burden index of the target lesion and the occurrence of slow TIMI flow.

## 2. Materials and Methods

### 2.1. Study Population

All eligible patients who underwent PCI for coronary artery disease with coronary NIRS-IVUS imaging at Korea University Hospital (Seoul, Korea) between April 2016 and June 2020 were prospectively enrolled in this study. Patients aged 19 years or older were eligible for enrolment, and the inclusion and exclusion criteria were as follows. For inclusion, patients who underwent PCI after performing target vessel NIRS-IVUS-imaging and who agreed to the study plan and clinical follow-up plan, voluntarily decided to participate in this clinical study, and agreed in providing written consent were enrolled. Patients were excluded from the study population if they (1) could not perform cardiovascular angiography due to severe symptoms of heart failure; (2) had an expected life expectancy of within 1 year due to accompanying disease; and (3) were women of childbearing age who planned to become pregnant within the study period. In total, 636 consecutive patients—and 636 vessels—were included in the analysis. NIRS-IVUS imaging was performed at the target vessel before PCI and afterwards under selected clinical settings, including patient stability. The decision to undergo NIRS-IVUS imaging was made at the time of angiography and solely by the operator. The study was approved by the ethics committee of the Korea University Hospital. Written informed consent was obtained from all the patients.

### 2.2. PCI and NIRS-IVUS

Angiography was performed according to standard methods, and the decision to perform PCI (stent insertion, drug-eluting ballooning, or simple ballooning) was made solely by the operator. All decisions regarding intervention were based on the latest guidelines for coronary revascularization [11,12]. Angiograms, NIRS-IVUS images, chemograms, and TIMI flow assessments were analyzed and adjudicated in an independent core laboratory.

A target vessel was selected according to the presence of significant stenosis, which was considered accountable for clinical manifestations. For patients with multivessel disease, the vessel with the most critical stenosis was selected as the target vessel for stable coronary artery disease and non-ST elevation acute coronary syndrome (ACS) patients, while ECG and clinical presentations were also considered in ST-elevation myocardial infarction (STEMI) patients. We performed target vessel assessment using NIRS-IVUS imaging for all target lesions before PCI, using an automated pullback system with combined NIRS and IVUS imaging (InfraReDx, Bedford, MA, USA). NIRS-IVUS imaging was performed before any intervention, including pre-ballooning or PCI, took place, unless indicated otherwise such as the patient’s clinical instability. NIRS identifies lipid-rich plaques by creating a color-coded chemogram marked with colors ranging from yellow to red, with yellow and red zones representing the highest and lowest probabilities of lipid core plaque (LCP) presence, respectively [9]. Block chemograms were analyzed to determine the extent of lipid core plaques, and the extent of lipid core burden (LCB) in the treatment zone was calculated as the maximal LCBI (maxLCBI) measured by NIRS for each of the 4 mm longitudinal segments in the treatment zone. The TIMI flow after the intervention was assessed and recorded.

### 2.3. Clinical Outcomes

The primary endpoint was the association between a decreased TIMI flow and LCBI. The secondary outcome of the study was the occurrence of adverse clinical events related to the TIMI flow grade. The incidence of major adverse cardiovascular events (MACE) comprised of cardiac death, myocardial infarction, stent thrombosis, and target lesion revascularization (TLR) was recorded during the follow-up period. Myocardial infarction (MI) was defined as elevation of levels of serum markers of myocardial necrosis (creatinine kinase-MB, troponin T, and troponin I) according to the guidelines, which included only spontaneous MI and not peri-procedural MI [13]. Stent thrombosis was defined according to the Academic Research Consortium (ARC) definitions and was only related to target lesions treated with PCI [14]. TLR was defined as any procedure or surgery on the coronary artery (PCI or coronary artery bypass grafting) that was not initially planned during the index angiography. Each patient had a follow-up visit 90 days after the index procedure, and/or before if clinically needed, for general clinical evaluation, including that of MACE occurrence. The presence and characteristics of MACE were determined by evaluating the inpatient and outpatient medical charts for each patient.

### 2.4. Statistics

Categorical variables were compared using Pearson’s χ^2^ with continuity correction or Fisher’s exact test. Continuous variables were compared using Student’s *t*-test. All categorical variables are reported as counts and percentage frequencies. Continuous variables are reported as mean ± standard deviation (SD) or median (interquartile range (IQR)). The Kaplan–Meier method was used to analyze and compare the cumulative event rate for the decreased and normal TIMI flow groups, for which the log-rank test was performed to show statistical significance.

Receiver-operating characteristic (ROC) analysis was performed to determine the relationship between LCBI and TIMI flow. Patients were dichotomized into “high” and “low” LCBI groups using ROC analysis and Youden index for the optimal cut-off point between the groups. For ROC analysis, we reported the area under the curve (AUC) and 95% confidence interval (CI). Multivariable logistic regression analysis was performed to compare TIMI flow between the high and low-LCBI groups, and the results were shown as odds ratios (OR) with 95% CI. Variables associated with the outcome (*p <* 0.2) in the univariable analysis were further analyzed in the multivariable model. For sensitivity analysis, an alternative cut-off level of ≥400 was used for LCBI based on previous retrospective studies [15,16]. All statistical tests were based on two-tailed tests; *p* < 0.05 was considered statistically significant. All analyses were performed using IBM SPSS Statistics 25.0 (IBM Corp., Armonk, NY, USA).

## 3. Results

### 3.1. Baseline and Lesion Characteristics

A total of 636 patients were divided into decreased (*n* = 90, TIMI less than 3) and normal (*n* = 546, TIMI 3) TIMI flow groups. The mean age of the patients was 65.5 ± 10.7 years, and 167 (26.3%) were female. Compared with the normal TIMI flow group, the decreased TIMI flow group was predominantly male and had a lower prevalence of hypertension. The presence of other underlying risk factors, such as diabetes mellitus, dyslipidemia, and congestive heart failure, did not differ between the two groups (Table 1). While ACS presentation as a factor did not differ significantly between the two TIMI flow groups, the comparison of clinical diagnoses (stable angina, unstable angina, NSTEMI, and STEMI) between the normal and decreased TIMI flow groups showed that the frequency of STEMI diagnosis was numerically higher in the decreased TIMI flow group (10.0% vs. 3.8%). However, the difference between the two groups, in terms of clinical diagnosis, was not statistically significant (*p* = 0.063). All patients were treated with dual antiplatelet drugs at initial loading doses and maintenance doses at discharge, unless clinically indicated otherwise. Statins and other medications were administered according to guidelines (Table 2). Table 3 shows the baseline lesion and procedural characteristics according to TIMI flow grade. Compared with the normal TIMI flow group, the decreased TIMI flow group had a high pre-PCI maxLCBI_4mm_ (300 (143–468) vs. 392 (211–592), *p =* 0.001), large reference vessel area (12.7 ± 4.5 vs. 14.1 ± 4.7, *p =* 0.002), and small minimal luminal diameter (0.8 ± 0.4 vs. 0.7 ± 0.4, *p =* 0.024) in terms of NIRS-IVUS analysis. No significant differences were found between the two groups in terms of the extent of coronary disease and device selection.

### 3.2. Association between TIMI Flow and maxLCBI_4mm_

There was a statistically significant relationship between maxLCBI_4mm_ and change in TIMI flow after PCI. In ROC analysis, the optimal cutoff value of LCBI for predicting decreased TIMI flow was 354 (AUC, 0.61 (95% CI, 0.54–0.67), *p =* 0.001; Supplementary Figure S1). Accordingly, the high-LCBI group was defined as having a maxLCBI_4mm_ value of ≥354. In the decreased TIMI group, a higher percentage of patients had high LCBI than that in the normal TIMI group (60.0% in the decreased TIMI group vs. 40.8% in the normal TIMI group, *p* = 0.001).

In the analysis of predictors of decreased TIMI flow at the target lesion before the procedure, current smoking status (OR, 1.76 (95% CI, 1.020–3.03), *p =* 0.042), high LCBI (OR, 2.17 (95% CI, 1.38–3.42), *p <* 0.001) and the presence of plaque attenuation (OR, 1.76 (95% CI, 1.12–2.76), *p* = 0.014) were associated with an increased risk of decreased TIMI flow in univariable analyses (Table 4), whereas female sex (OR, 0.31 (95% CI, 0.15–0.61), *p =* 0.001) and hypertension were associated with a decreased risk of decreased TIMI flow (OR, 0.62 (95% CI 0.40–0.97), *p =* 0.036). In multivariable analysis, presence of left main (LM) disease (OR, 4.88 (95% CI, 1.10–21.6), *p =* 0.037), high LCBI (OR, 2.59 (95% CI, 1.33–5.04), *p =* 0.005), and the presence of attenuation (OR, 2.71 (95% CI 1.42–5.18), *p* = 0.003) remained significant predictors of decreased TIMI flow. Female sex (OR, 0.15 (95% CI, 0.05–0.52), *p =* 0.003), hypertension (OR, 0.51 (95% CI, 0.27–0.95), *p =* 0.034), and minimal post-PCI luminal diameter (OR, 0.30 (95% CI 0.10–0.90), *p* = 0.032) were inversely associated with decreased TIMI flow.

**Figure 1:**
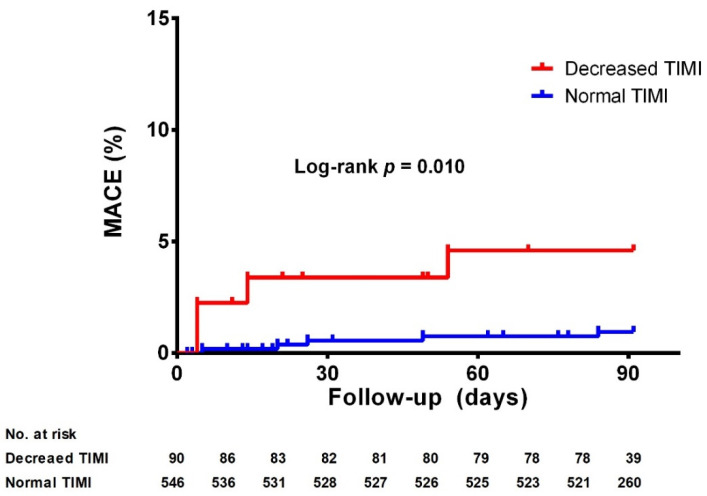
Kaplan–Meier curves for TIMI flow and the occurrence of major adverse cardiovascular events (MACE). Decreased post-PCI TIMI flow is associated with a significantly higher rate of MACE. TIMI, thrombolysis in myocardial infarction.

Additionally, for the analysis of the secondary endpoint, the incidence of MACE according to TIMI flow grade was evaluated, as illustrated by the Kaplan–Meier curves in Figure 1. During the 90-day follow-up period, the decreased TIMI flow group showed a higher incidence of MACE than the normal TIMI flow group (5.6% vs. 1.5%, log-rank *p =* 0.010), for which TLR was the main component (5.7% in decreased TIMI group vs. 1.3% in normal TIMI group, log-rank *p* = 0.005).

## 4. Discussion

The present study investigated the association between post-coronary interventional slow TIMI flow and the high-lipid core burden of the target lesion. The main findings of this study are as follows: First, decreased TIMI flow was more prevalent in patients with high pre-PCI target lesion-LCBI. Moreover, pre-PCI high lipid burden, expressed as maxLCBI_4mm_, measured using NIRS-IVUS imaging, is an independent predictor of decreased post-PCI TIMI flow for the target lesion. Second, a decreased TIMI flow in the target lesion was associated with an increased risk of MACE. To the best of our knowledge, the present study is the first to predict slow TIMI flow based on the lipid plaque burden, which is one of the characteristics of vulnerable plaques.

Incomplete flow restoration of an infarct-related coronary artery is a predictor of adverse outcomes such as reduced ventricular function and increased mortality in ACS [1,2,17,18] or chronic coronary syndrome patients [12]. The present study revealed that the decreased TIMI flow group had worse clinical outcomes compared to the normal TIMI flow group, which is consistent with previous reports [17,18]. However, despite knowing that decreased TIMI flow is associated with worse clinical outcomes, few predictors have been reported for decreased TIMI flow before coronary intervention. In this study, an association between a high pre-PCI maxLCBI_4mm_ of the target lesion and decreased TIMI flow after coronary intervention was observed. Furthermore, even after the multivariable analysis, high LCBI was an independent predictor of decreased TIMI flow after coronary intervention. Additionally, LM disease was an independent predictor of decreased TIMI flow, whereas female sex and hypertension was associated with a decreased risk of slow TIMI flow. Female sex was associated with higher post-PCI TIMI flow (≥2) in a prospective study of STEMI patients, with an adjusted OR of 0.52 for TIMI ≤ 1 [19]. In contrast, female sex was associated with postprocedural TIMI flow grade 0 to 2 with an OR of 1.68 in another prospective cohort study of STEMI patients [20]. We have found in our study that female sex was associated with decreased TIMI flow, but it thus seems from previous evidence that there is no definitive conclusion as to whether male or female sex acts as a protective or adverse factor in coronary blood flow. However, some evidence exists for estrogen levels exerting influence on coronary vasomotor behavior, which may explain the sex differences in TIMI flow [21]. The association of decreased TIMI flow with LM disease may be explained by the fact that the left main coronary artery (LMCA) is inherently larger than other coronary vessels, predisposing vessels with its involvement to a high plaque burden and thrombotic risk. Previous studies have shown that an increase in area stenosis suggests increased circumferential plaque burden [22]. A previous study has also shown that the presence of LMCA disease may indicate additive involvement of other coronary arteries, suggesting an increased diffuse atherosclerotic burden of the vessels [23]. Patients diagnosed with hypertension are more likely to already be on cardioprotective medications such as angiotensin II receptor blockers/angiotensin-converting enzyme inhibitors, calcium channel blockers and beta-blockers. Previous studies have reported that pre-treatment with such agents has preventive effects against the no-reflow phenomenon [24].

Various characteristics of atheromatous plaques constitute plaque vulnerability, including the degree of inflammation, thickness of fibrous caps, size of atheroma, and extent of calcification [25,26]. The lipid-rich core in an atheromatous plaque is often subject to rupture, potentially causing sudden thrombosis leading to ACS [25]. Postmortem studies on plaque histopathology have shown that ruptured plaques have larger necrotic cores with higher lipid contents, as assessed by the degree of luminal narrowing and the number of lipid-laden cells or cholesterol clefts within the core [25]. Although lipid-rich core plaques are more prevalent in the target lesions of ACS patients, they are also present in chronic coronary syndrome patients [27]. Theoretically, rupture of lipid-rich and inflamed plaques may cause spontaneous microembolization of plaque material and obstruction of coronary microcirculation [28]. Additionally, intervention of the lipid-rich culprit lesion may even promote microembolism by shedding the thrombotic layer that covers the ruptured plaque [28]. In the context of this study, lesions with lipid-rich core plaques bear a greater risk of suboptimal revascularization and adverse outcomes. However, the identification of lipid-rich core plaques using traditional intravascular imaging modalities including IVUS and OCT is limited.

NIRS-IVUS uses the differential properties of substances to absorb and scatter near-infrared light to detect lipid core plaques. A recent prospective cohort study on lipid-rich plaques revealed that patients with a high-lipid-core burden had a higher incidence of subsequent adverse events [29]. These observations suggest that additional information on plaque composition gathered through NIRS-IVUS imaging could predict the potential occurrence of subsequent MACE. Recently, the PROSPECT II study indicated that patients with large lipid-rich cores and large plaque burdens detected using NIRS-IVUS imaging had an increased risk of future adverse cardiac outcomes [7]. However, all the above-mentioned studies have addressed the association between lipid-rich cores of non-target lesions and clinical outcomes. That is, the association between the large lipid-rich cores of target lesions and outcomes has been poorly elucidated. The present study showed that large lipid-rich cores of target lesions were associated with decreased TIMI flow which consequently led to worse clinical outcomes. Additionally, a recent study by Terada et al. reported that in STEMI patients, high LCBI values from NIRS-IVUS imaging were associated with microvascular obstruction, which supports our findings in that high lipid burden leads to myocardial damage [30].

Previous NIRS studies have revealed that larger lipid core plaques, defined as those with higher maxLCBI_4mm_ values, were significantly associated with subsequent adverse clinical outcomes at different thresholds [15,16,31]. However, the previous studies were small observational studies. Recently, two large-scale prospective analyses were conducted to investigate the use of cut-off values as independent predictors of adverse events. The Lipid-Rich Plaque study and PROSPECT II trials suggested using maxLCBI_4mm_ cut-offs of 400 and 324.7, respectively, as independent predictors of subsequent adverse events [7,29]. In the present study, the optimal cut-off value of maxLCBI_4mm_ for predicting decreased TIMI flow was 354. Based on the cut-off value, a high LCBI group was associated with decreased TIMI flow in the multivariable analysis. Additionally, even when using a cut-off maxLCBI value from previous studies, a high LCBI group was consistently associated with decreased TIMI flow in the multivariable analysis (Supplementary Table S1). These observations suggest that operators should be cautious about the higher risk of decreased TIMI flow when conducting PCI for target lesions with higher maxLCBI values.

Our study has several limitations. First, it was based on retrospective analysis of a single-center registry. Thus, despite adjusting for variables to reduce confounding factors, the present study has the inherent limitations of unmeasured confounding factors such as reperfusion time in STEMI patients. Second, the total number of events for MACE was relatively small. Correspondingly, the majority of MACE in this study were due to target lesion revascularization. Therefore, it raises the possibility that procedure-related factors, rather than slow blood flow, contributed more to the occurrence of MACE. Further investigation with an evaluation of a larger sample population is therefore necessary to consolidate our findings for clinical implications. Third, the AUC for the ROC analysis between maxLCBI4 mm and the decrease in TIMI flow was not as high as expected, but the presence of a statistically significant predictability suggests that LCBI is one of the factors that influences slow TIMI flow. Additionally, several previous studies have revealed that high LCBI values are associated with poor clinical outcomes, especially in terms of MACE. However, no direct relationship was shown between LCBI and TIMI flow during the procedure. This relationship potentially provides a basis for the management of lesions according to LCBI values, both in terms of whether to perform PCI for angiographically intermediate but high-LCBI lesions, and taking preventive measures during PCI of high-LCBI lesions. As the focus of this study was on the relationship between the high-lipid composition of plaques and the occurrence of no-reflow during PCI, a direct relationship between plaque composition and clinical outcome should be explored in future studies. Fourth, as pre-dilations or thrombo-aspirations may cause distal embolization, NIRS-IVUS imaging was performed before any intervention was implemented. However, securing distal flow was prioritized under circumstances of total occlusion, especially for STEMI patients, which may have influenced the post-PCI TIMI flow in such patients.

## 5. Conclusions

In the present study, a high maxLCBI_4mm_ of the target lesion, as measured using NIRS-IVUS imaging, was an independent predictor of slow TIMI flow following PCI. Performing PCI in high-LCBI lesions, therefore, may necessitate adjunctive measures to prevent suboptimal post-PCI reperfusion.

## Figures and Tables

**Table 1:** Baseline clinical characteristics of the study population.

	Total	Normal TIMI	Decreased TIMI	*p*-Value
	(*n* = 636)	(*n* = 546)	(*n* = 90)	
Sex (female)	167 (26.3%)	157 (28.8%)	10 (11.1%)	0.001
Age (years)	65.5 ± 10.7	65.5 ± 10.5	65.7 ± 11.5	0.876
Hypertension	389 (61.2%)	343 (62.8%)	46 (51.1%)	0.035
Diabetes mellitus	205 (32.2%)	184 (33.7%)	21 (23.3%)	0.052
Dyslipidemia	116 (18.2%)	105 (19.2%)	11 (12.2%)	0.140
Congestive heart failure	27 (4.2%)	22 (4.0%)	5 (18.5%)	0.506
LV EF (%)	55.7 ± 8.5	55.9 ± 8.4	54.3 ± 8.9	0.122
Current smoker	129 (20.3%)	104 (19.0%)	25 (27.8%)	0.123
Family Hx. of CAD	72 (11.3%)	65 (11.9%)	7 (7.8%)	0.252
Hx. of PCI	68 (10.7%)	60 (11.0%)	8 (8.9%)	0.713
Hx of CABG	7 (1.1%)	7 (1.3%)	0 (0.0%)	1.000
Hx of CVA	78 (12.3%)	66 (12.1%)	12 (14.2%)	0.739
Clinical presentation				0.063
Stable angina	308 (48.4%)	270 (49.5%)	38 (42.2%)	
Unstable angina	249 (39.2%)	215 (39.4%)	34 (37.8%)	
NSTEMI	49 (7.7%)	40 (7.3%)	9 (10.0%)	
STEMI	30 (4.7%)	21 (3.8%)	9 (10.0%)	
Presentation as ACS	328 (51.6%)	276 (50.5%)	52 (57.8%)	0.204
Peak CK-MB (ng/mL)	3.50 (2.10–9.38)	3.28 (2.03–7.79)	8.29 (3.10–50.60)	<0.001

Values are presented as mean ± SD, median (IQR) or *n* (%). Analysis by χ^2^ test with continuity correction or Fisher’s exact method. ACS, acute coronary syndrome; CABG, coronary artery bypass graft; CAD, coronary artery disease; CVA, cerebrovascular accident; LVEF, left ventricular ejection fraction; NSTEMI, non-ST elevation myocardial infarction; PCI, percutaneous coronary intervention; STEMI, ST-elevation myocardial infarction; TIMI, thrombolysis in myocardial infarction.

**Table 2:** Discharge medications of the study population.

	Total	Normal TIMI	Decreased TIMI	*p*-Value
	(*n* = 636)	(*n* = 546)	(*n* = 90)	
Dual antiplatelet therapy	573 (90.1%)	489 (89.6%)	84 (93.3%)	0.358
ACEI/ARB	335 (52.7%)	291 (53.3%)	44 (48.9%)	0.508
BB	238 (37.4%)	194 (35.5%)	44 (48.9%)	0.021
CCB	255 (40.1%)	230 (42.1%)	25 (27.8%)	0.014
Diuretics	78 (12.3%)	69 (12.6%)	9 (10.0%)	0.594
Statins	596 (93.7%)	508 (93.0%)	88 (97.8%)	0.139
Ezetimibe	175 (27.5%)	158 (28.9%)	17 (18.9%)	0.064

Values are presented as *n* (%). Analysis by χ^2^ test with continuity correction or Fisher’s exact method. ACEI, Angiotensin-converting enzyme inhibitors; ARB, Angiotensin receptor blockers; BB, beta-blockers; CCB, calcium channel blockers; TIMI, thrombolysis in myocardial infarction.

**Table 3:** Procedural characteristics of study population.

	Total	Normal TIMI	Decreased TIMI	*p*-Value
	(*n* = 636)	(*n* = 546)	(*n* = 90)	
Extent of coronary disease				0.132
0-vessel disease	3 (0.5%)	2 (0.4%)	1 (1.1%)	
1-vessel disease	289 (45.4%)	241 (44.1%)	48 (53.3%)	
2-vessel disease	196 (30.8%)	175 (32.1%)	20 (22.2%)	
3-vessel disease	148 (23.3%)	127 (23.3%)	21 (23.3%)	
LM disease	92 (14.5%)	84 (15.4%)	8 (8.9%)	0.144
Device selection				
Balloon diameter	2.5 ± 0.4	2.5 ± 0.4	2.45 ± 0.3	0.627
Stent diameter	3.4 ± 1.2	3.3 ± 1.3	3.5 ± 0.6	0.302
Stent length	22.2 ± 7.4	22.1 ± 7.4	22.9 ± 6.8	0.384
Post-balloon diameter	3.5 ± 0.5	3.5 ± 0.5	3.6 ± 0.7	0.099
IVUS parameter				
Reference diameter	3.6 ± 0.6	3.6 ± 0.6	3.7 ± 0.7	0.174
Reference vessel area	12.7 ± 4.5	12.7 ± 4.5	14.1 ± 4.7	0.002
Minimal luminal diameter	0.8 ± 0.4	0.8 ± 0.4	0.7 ± 0.4	0.024
Minimal lumen area	3.0 ± 6.6	3.0 ± 7.1	2.9 ± 1.3	0.854
Area of stenosis	77.6 ± 13.0	77.6 ± 12.2	78.0 ± 17.1	0.749
Lesion length	21.2 ± 11.2	21.0 ± 11.3	22.7 ± 10.6	0.190
Minimal stent diameter	3.1 ± 0.6	3.1 ± 0.6	3.2 ± 0.6	0.113
Target lesion LCBI				
maxLCBI_4mm_ (pre PCI)	317 (154–484)	300 (143–468)	392 (211–592)	0.001
maxLCBI_4mm_ (post PCI)	53 (0–193)	49 (0–187)	78 (0–243)	0.316

Values are presented as mean ± SD, median (IQR) or *n* (%). IVUS, intravascular ultrasound; LCBI, lipid core burden index; LM, left main; TIMI, thrombolysis in myocardial infarction.

**Table 4:** Association between TIMI flow and clinical characteristics.

	Univariable			Multivariable		
	OR	95% CI	*p*-Value	OR	95% CI	*p*-Value
Clinical characteristics						
Female sex	0.31	0.15–0.61	0.001	0.15	0.05–0.52	0.003
Age	1.00	0.98–1.02	0.875			
Hypertension	0.62	0.40–0.97	0.036	0.51	0.27–0.96	0.038
Diabetes mellitus	0.60	0.36–1.00	0.053	0.69	0.32–1.46	0.329
Dyslipidemia	0.589	0.30–1.14	0.114	1.00	0.40–2.51	0.994
Current smoker	1.76	1.02–3.03	0.042	1.89	0.86–4.18	0.114
Congestive heart failure	1.40	0.52–3.80	0.510			
Hx of CVD	1.12	0.58–2.17	0.739			
Hx of PCI	0.79	0.36–1.71	0.551			
Hx of CABG	0.00					
FHx of CAD	0.62	0.28–1.41	0.256			
Presentation as ACS	1.34	0.85–2.10	0.205			
Angiographic characteristics						
Complex lesion	0.70	0.44–1.10	0.081	0.60	0.30–1.21	0.152
LM disease	1.86	0.87–3.99	0.109	4.88	1.10–21.6	0.037
pre-PCI maxLCBI_4mm_ ≥ 354	2.17	1.38–3.42	0.001	2.59	1.33–5.04	0.005
Device selection						
Balloon diameter	0.90	0.38–2.11	0.811			
Balloon length	1.18	1.06–1.31	0.003	1.12	0.98–1.28	0.092
Stent diameter	1.07	0.93–1.23	0.350			
Stent length	1.05	0.98–1.01	0.384			
Post-balloon diameter	1.57	0.97–2.54	0.066	1.37	0.51–3.64	0.528
Post-balloon length	1.01	0.93–1.10	0.744			
IVUS parameter						
Reference diameter	1.27	0.90–1.81	0.175	1.03	0.43–2.45	0.953
Minimal luminal diameter	0.46	0.24–0.90	0.024	0.30	0.10–0.90	0.032
Minimal lumen area	1.00	0.96–1.04	0.862			
Area of stenosis	1.04	1.01–1.07	0.003	1.07	1.00–1.15	0.065
Lesion length	1.01	0.99–1.03	0.192			
Minimal stent diameter	1.34	0.92–1.97	0.131	0.91	0.32–2.60	0.859
Presence of plaque attenuation	1.76	1.12–2.76	0.014	2.71	1.42–5.18	0.003

Univariable and multivariable analyses by logistic regression. OR, odds ratio; ACS, acute coronary syndrome; CABG, coronary artery bypass graft; CAD, coronary artery disease; CVA, cerebrovascular accident; IVUS, intravascular ultrasound; LCBI, lipid core burden index; PCI, percutaneous coronary intervention; TIMI, thrombolysis in myocardial infarction.

## Data Availability

The data generated in this study are available from the corresponding author upon reasonable request.

## Supplementary Materials

The following supporting information can be downloaded at: https://www.mdpi.com/article/10.3390/jcm11185401/s1, Figure S1: Receiver-operating characteristic curve for maxLCBI_4mm_ and TIMI flow; Table S1: Association between TIMI flow and clinical characteristics, using LCBI cut-off as ≥400.
