# Supplementary material for: Association between High Lipid Burden of Target Lesion and Slow TIMI Flow in Coronary Interventions
Source: J Clin Med. 2022 Sep 14;11(18):5401. doi: 10.3390/jcm11185401 (PMC9502085; doi:10.3390/jcm11185401)
Supplement: Supplementary file 1 [file jcm-11-05401-s001.zip › jcm-1889600-supplementary.pdf]

Supplementary Materials

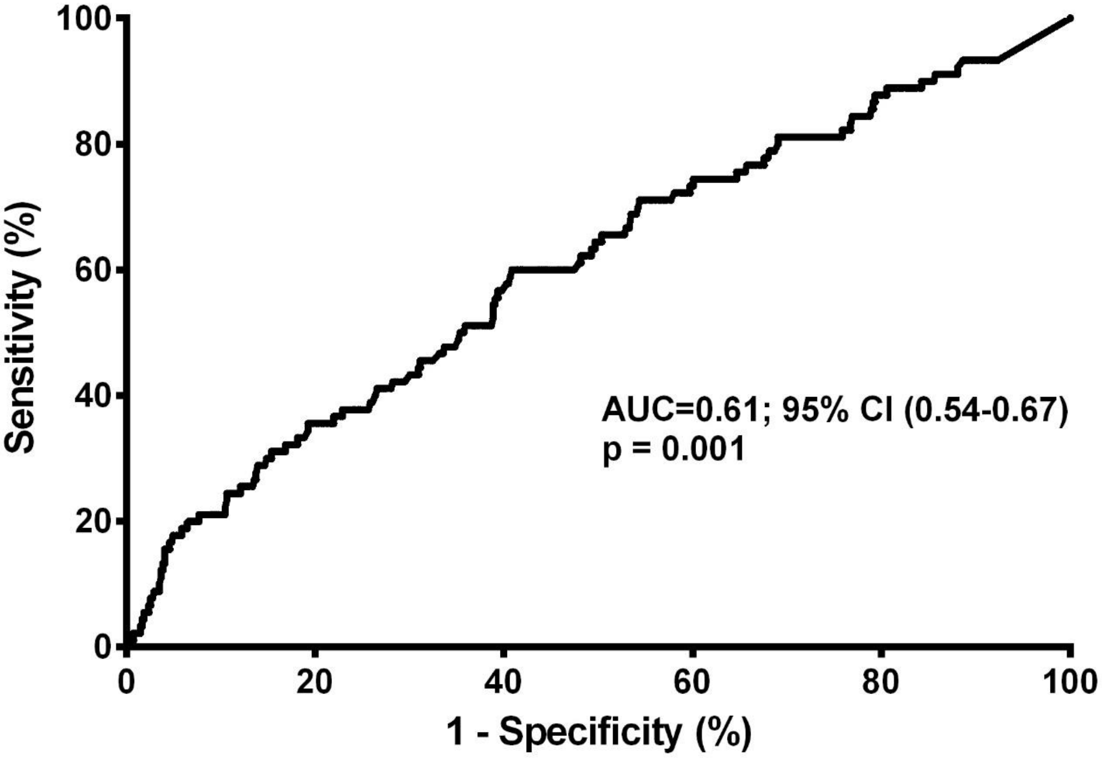

Supplementary Figure S1. Receiver-operating characteristic curve for maxLCBI4mm and TIMI flow. maxLCBI$_{4\ mm}$: maximal lipid core burden index over any 4 mm length TIMI: thrombolysis in myocardial infarction

Supplementary Table S1. Association between TIMI flow and clinical characteristics, using LCBI cut-off as ≥400.

	Univariable			Multivariable		
	**OR**	**95% CI**	***p*-Value**	**OR**	**95% CI**	***p*-Value**
**Clinical characteristics**						
Male sex	3.23	1.63-6.39	0.001	5.55	1.54-19.8	0.009
Age	1.00	0.98-1.02	0.875	1.02	0.98-1.05	0.364
Hypertension	0.62	0.40-0.97	0.036	0.49	0.25-0.95	0.034
Diabetes	0.60	0.36-1.00	0.053	0.66	0.32-1.39	0.277
Dyslipidemia	0.589	0.30-1.14	0.114	1.13	0.46-2.79	0.79
Current smoker	1.76	1.02-3.03	0.042	1.58	0.71-3.53	0.266
Congestive heart failure	1.40	0.52-3.80	0.510			
Hx of CVD	1.12	0.58-2.17	0.739			
Hx of PCI	0.79	0.36-1.71	0.551			
Hx of CABG	0.00					
FHx of CAD	0.62	0.28-1.41	0.256			
Presentation as ACS	1.34	0.85-2.10	0.205			
**Angiographic characteristics**						
Complex lesion	0.70	0.44-1.10	0.081	0.63	0.32-1.22	0.169
LM disease	1.86	0.87-3.99	0.109	4.99	1.09-22.8	0.038
pre-PCI maxLCBI4mm ≥400	1.74	1.11-2.73	0.016	2.21	1.14-4.29	0.019
**Device selection**						
Balloon diameter	0.90	0.38-2.11	0.811			
Balloon length	1.18	1.06-1.31	0.003	1.09	0.95-1.24	0.224
Stent diameter	1.07	0.93-1.23	0.350			
Stent length	1.05	0.98-1.01	0.384			
Post-balloon diameter	1.57	0.97-2.54	0.066	1.50	0.60-3.74	0.385
Post-balloon length	1.01	0.93-1.10	0.744			
**IVUS parameter**						
Reference diameter	1.27	0.90-1.81	0.175	1.32	0.58-3.03	0.509
Minimal luminal diamete	0.46	0.24-0.90	0.024	0.248	0.08-0.81	0.021
Minimal lumen area	1.00	0.96-1.04	0.862			
Area of stenosis	1.04	1.01-1.07	0.003	1.06	0.96-1.16	0.241
Lesion length	1.01	0.99-1.03	0.192	1.00	0.98-1.03	0.837
Minimal stent diameter	1.34	0.92-1.97	0.131	0.81	0.29-2.24	0.685
